# Efficacy and safety of *Ginkgo biloba* extract as an adjuvant in the treatment of Chinese patients with sudden hearing loss: a meta-analysis

**DOI:** 10.1080/13880209.2023.2190782

**Published:** 2023-03-31

**Authors:** Chao Yuan, Huan Zhang, Cuicui Sun, Kai Zhang

**Affiliations:** a Department of Pharmacy, Weifang People’s Hospital, Weifang, China; b Department of Pharmacy, Henan NO.3 Provincial People’s Hospital, Zhengzhou, China; c Department of Clinical Pharmacy, Qilu Hospital of Shan Dong University, Jinan, China; d Department of Gastroenterology, Weifang People’s Hospital, Weifang, China

**Keywords:** GBE, SHL, total effective rate, cure rate, pure tone hearing threshold, hemorheology, randomized controlled trials

## Abstract

**Context:**

*Ginkgo biloba* Linn (Ginkgoaceae) [leaves extract (GBE)] is authorized for the treatment of sudden hearing loss (SHL); however, its clinical feasibility in SHL has not been thoroughly investigated.

**Objective:**

To evaluate the efficacy and safety of adjuvant GBE in the treatment of SHL.

**Materials and methods:**

We used PubMed, EMBASE, Web of Science, Cochrane Library, China National Knowledge Infrastructure, Wanfang, Chinese Scientific Journal Database, China Biomedical Database for literature research, starting from inception to 30 June 2022. The key terms: *Ginkgo biloba* extract, Sudden Sensorineural Deafness. This meta-analysis contained randomized controlled trials that compared the safety and efficacy of the combination of GBE and general treatments (GT) with GT alone for SHL. The extracted data were analyzed using Revman5.4 software with risk ratio (RR), 95% confidence intervals (CI) and mean difference (MD).

**Results:**

Our meta-analysis included 27 articles with a total of 2623 patients. The results revealed that the effects of GBE adjuvant therapy was superior than GT (total effective rate: RR = 1.22, 95% CI: 1.18–1.26, *p* < 0.00001), the pure tone hearing threshold (*MD* = 12.29, 95% CI: 11.74–12.85, *p* < 0.00001) and hemorheology indexes (whole blood high shear viscosity: *MD* = 1.46, 95% CI: 0.47–2.44, *p* = 0.004) after treatment were significantly improved compared to non-treatment, while there was no significant difference as for hematocrit (red blood cells) (*MD* = 4.15, 95% CI: −7.15–15.45, *p* = 0.47).

**Conclusion:**

The efficacy of GBE + GT for the treatment of SHL may be more promising than GT alone.

## Introduction

Sudden hearing loss (Byl 1984; O’Malley and Harnes 2008; Wen et al. 2020; Wang and Ma 2022), known as sudden sensorineural hearing loss, is a common emergency in otolaryngology. Its clinical symptoms include unilateral hearing loss, accompanied by tinnitus, dizziness, nausea and others. Although the etiology and pathogenesis of SHL are still not clear until now, some researchers (Yu and Yang 2015; Beckers et al. 2021; Ricciardiello et al. 2021; Si, Liu, et al. 2022) attribute it to viral infection, circulatory system dysfunction, immune dysfunction, etc. There is still no standard treatment strategy; treatment for sudden deafness is basically comprehensive, and the application of drugs in nutritive nerve and circulatory improvements is common (Li et al. 2016; Chandrasekhar et al. 2019).

*Ginkgo biloba* Linn (Ginkgoaceae) leaves extract (GBE) is an active ingredient extracted from the dried leaves of *Ginkgo biloba* (Yang et al. 2016; Barth et al. 2021), with the effects of promoting blood circulation and removing blood stasis, activating the collaterals to relieve pain, warming the lungs and relieving asthma, removing turbidity and reducing lipid. It is widely used for the collateral obstruction, chest paralysis and heartache, stroke hemiplegia (Zhang et al. 2021). GBE contains *Ginkgo* flavonoids, quercetin, ginkgolides, organic acids, and other components (Wang et al. 2022), which exert a variety of pharmacological effects, such as dilating blood vessels, regulating blood lipids, antagonizing platelet activating factor, protecting ischemic injury, anti-inflammatory, and antitumor (Liu et al. 2014). It also has definite curative effects for the treatment of coronary heart disease, cerebral thrombosis, cerebral ischemia, cerebral dysfunction, sequelae of brain trauma, nervous system diseases and scavenging oxygen free radicals (Gai et al. 2017; Liu et al. 2021; Xiao et al. 2022).

GBE has the effect of improving inner ear circulation (Kandiah et al. 2021), which is suitable for the diagnosis and treatment of tinnitus, vertigo, hearing loss and neurological disorders. Some studies (Gao et al. 2015; Zeng and Liu 2020; Qu and Gao 2021) have shown that it also has a specific therapeutic effect on SHL, but the sample size included in each study is small, and such experimental results are not objective and comprehensive, so it impacts on the reliability of the combination effect and the scope of the clinical promotion and application is not ideal. We performed this meta-analysis to evaluate the efficacy, safety of GBE, and provided scientific evidence to the design and implementation of SHL and scientific basis for its clinical practice.

## Materials and methods

### Search strategy

The literature search was conducted in database, including PubMed, EMBASE, Web of Science, Cochrane Library, China National Knowledge Infrastructure (CNKI), Wanfang, Chinese Scientific Journal Database (VIP), China Biomedical Database (CBM), and the following keywords were used to obtain the randomized clinical trials (RCTs): *Ginkgo biloba* extract, Ginkgo leaf extract, GBE 761, *Ginkgo biloba* extract 761, Sudden Deafness, Sudden Sensorineural Deafness, Idiopathic Deafness, Deafness sudden, Sudden hearing loss, Sudden deafness, etc. The date was from the establishment of database to 30 June 2022.

### Eligibility criteria

#### Inclusion criteria

1. It conformed to the diagnostic criteria in the SHL Diagnosis and Treatment Guidelines of the Chinese Medical Association (IOM [Institute of Medicine] 2006).
2. Randomized controlled trials (RCTs) involving patients diagnosed with SHL.
3. The participants had not previously taken *Ginkgo biloba* extract, and not participated in relaxation training and psychotherapy, with normal communication skills.
4. Research compared the clinical results of the combination of GBE and general treatments (experimental group) with general treatments alone (control group).

#### Exclusion criteria

1. Research about SHL complicated with other diseases.
2. The standard for the experimental group or the control group is not suitable; the experimental group also contains general treatment measures not in the control group.
3. The data is vague, incomplete or not be extracted.
4. Non-clinical studies, review papers, meta-analyses, meeting abstracts, case reports and dissertations.

### Outcome definitions

The main outcomes were the total effective rate and cure rate, and the secondary outcomes were pure tone hearing threshold, hemorheology and adverse drug reactions (ADR). The cure standard was that the patients with SHL basically to the original level. The ineffectiveness means that the patients with SHL had no obvious improvement or further deterioration. Total effective rate = (number of all cases- number of ineffective cases)/the total number of patients × 100%. The cure rate = the number of cured cases/the total number of patients × 100%.

### Data extraction and quality assessment

The following data were extracted from eligible literature: the first author; year of publication; number of cases; age of patient; intervention; GBE dosage; duration of treatment and ADR.

The final articles included in our meta-analysis were independently screened by two reviewers. The titles and abstracts of the remaining researches were reviewed after excluding duplicate studies. The full text of the remaining studies was independently reviewed by two reviewers. If two reviewers disagreed on the article that is eventually included, a third reviewer will resolve the dispute.

According to the bias risk assessment recommended by Revman5.4, the evaluation criteria included seven domains of evaluation: (a) random sequence generation (selection bias); (b) allocation concealment (selection bias); (c) blinding of participants and personnel (performance bias); (d) blinding of outcome assessment (detection bias); (e) incomplete outcome data (attrition bias); (f) selective reporting (reporting bias); (g) other bias.

The Cochrane Correspondence Network RCT was used to assess each project in terms of low risk (+), unknown risk (?) and high risk (−). The quality evaluation of retrieved studies was conducted by group discussion.

### Statistical methods

Revman5.4 software was used for this meta-analysis. The relative risk (RR) and its 95% confidence interval (CI) of dichotomous data were used as the effect analysis statistics, and the variations in pure tone hearing threshold and hemorheology were expressed as mean difference (*MD*) with 95% CI of continuous data. *I*^2^ statistics and chi-square test were used to assess the statistical heterogeneity. Values of *p* > 0.1 or *I*^2^ < 50% denoted the existence of low heterogeneity, the fixed-effect model was chosen; otherwise, the random-effects model was adopted. The *p*-value less than 0.05 was considered statistically significant. A funnel plot was used to test for publication bias.

## Results

### Search results

**Figure 1.**
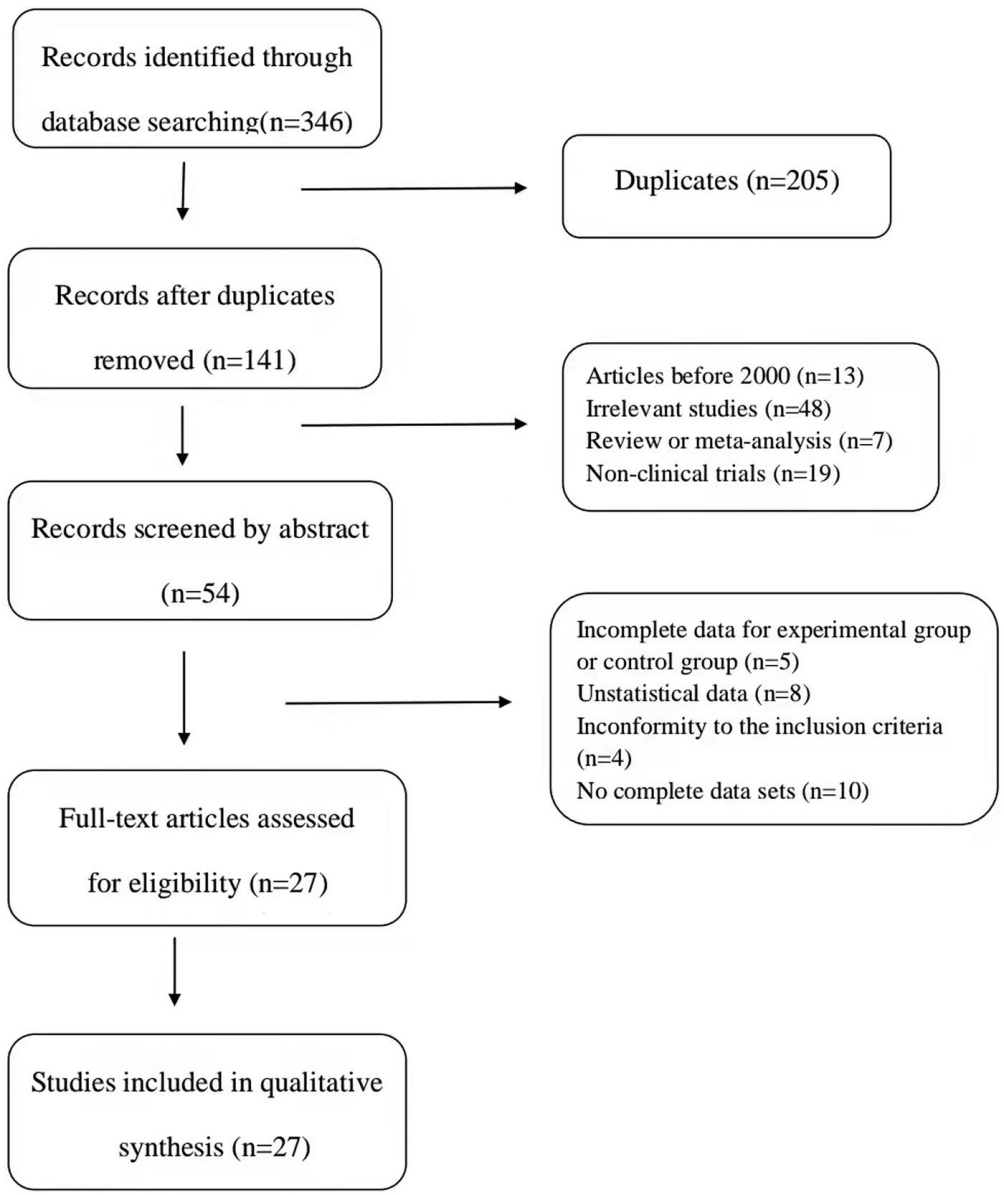
Study selection process for the meta-analysis.

According to the search strategy, a total of 346 relevant studies were included in this analysis. Among them, 5 articles were included in PubMed, 2 articles in EMBASE, 12 articles in Web of Science, 3 articles in Cochrane Library, 85 articles in CBM, 65 articles in CNKI, 98 articles in WanFang, and 76 articles in VIP. After removing duplicate articles and research, 141 articles were retained. Then, 87 articles were excluded by reviewing the titles and abstracts. Subsequently, 27 articles were excluded after assessment of the full text, on account of the following: incomplete data for experimental group or control group (*n* = 5), no statical data (*n* = 8), no complete data sets (*n* = 10), inconformity to the inclusion criteria (*n* = 4), finally 27 eligible articles (Wang and Li 2008; Su 2013; Han et al. 2015; Liang et al. 2017; Shi 2017; Wang 2017; Abu et al. 2018; Fu 2018; Liu et al. 2018; Meng et al. 2018; Xie 2018; Ye et al. 2018; Yu and Xu 2018; Zhao et al. 2018; Zhang GJ et al. 2019; Zhang HJ et al. 2019; Zhang JY et al. 2019; Dong 2020; Liang 2020; Lu et al. 2020; Xin et al. 2020; Huang and Luo 2021; Jia et al. 2021; Liu 2021; Tang 2021; Wu and Run 2021; Sun 2022) were included in our meta-analysis (Figure 1).

### Patient characteristics

All 27 studies with a total of 2623 patients of SHL were RCTs, including 1312 patients in the experimental group and 1311 patients in the control group. The experimental group was treated with *Ginkgo biloba* extract combined with other methods, and the control group was treated with other basic treatments. Randomization was mentioned in all 27 studies, and the sample size of a single RCT was 100 at most and 15 at least. *Ginkgo biloba* tincture (Beijing China Resources High-tech Natural Medicine Co., Ltd.) was mentioned in 2 studies (Fu 2018; Yu and Xu 2018), *Ginkgo biloba* extract injection (Taiwan Jisheng Chemical Pharmaceutical Co., Ltd.) in 10 studies (Han et al. 2015; Abu et al. 2018; Meng et al. 2018; Ye et al. 2018; Zhang et al. 2019; 2019; Dong 2020; Lu et al. 2020; Xin et al. 2020; Huang and Luo 2021), *Ginkgo biloba* extract injection (YOUCARE Pharmaceutical Group Co., Ltd.) in 7 studies (Liang et al. 2017; Wang 2017; Zhao et al. 2018; Jia et al. 2021; Liu 2021; Tang 2021; Wu and Run 2021), while no specific information of GBE was described in 7 studies (Wang and Li 2008; Su 2013; Shi 2017; Xie 2018; Zhang et al. 2019; Liang 2020; Sun 2022). The characteristics of the studies that are included in this meta-analysis are summarized in Table 1.

**Table 1.** Characteristics of the included studies.

Included studies	Simple size (Exp /Con)	Age	Interventions measure (Exp vs Con)	GBE dosage	Time	Outcomes
Abu et al. 2018	43/43	45–56	GBE + GT vs GT	87.5 mg/day	10 days	T,C,A
Dong 2020	43/43	16–70	GBE + GT vs GT	105 mg/day	14 days	T,A
Fu 2018	50/50	NG	GBE + GT vs GT	6 mL/day	10 days	T,A
Han et al. 2015	62/62	36–62	GBE + GT vs GT	105 mg/day	10 days	T
Huang and Luo 2021	26/26	20–59	GBE + GT vs GT	105 mg/day	10 days	T,C,A
Jia et al. 2021	50/50	40–54	GBE + GT vs GT	87.5 mg/day	10 days	T,C,A
Liang et al. 2017	51/51	18–72	GBE + GT vs GT	70–140 mg/day	14 days	T,C,A
Liang 2020	20/20	66–80	GBE + GT vs GT	40 mL	14 days	T,A
Liu et al. 2018	31/31	18–64	GBE + GT vs GT	20 mL	7–10 days	T,C
Liu 2021	43/43	20–63	GBE + GT vs GT	70–140 mg/day	14 days	T
Lu et al. 2020	48/48	30–58	GBE + GT vs GT	70–140 mg/day	14 days	T,C
Meng et al. 2018	64/64	32–77	GBE + GT vs GT	87.5 mg/day	10 days	T,C,A
Shi 2017	100/100	37–48	GBE + GT vs GT	87.5 mg/day	10 days	T,C,A
Su 2013	42/42	13–65	GBE + GT vs GT	15 mL	1–7 days	T,C,A
Sun 2022	15/15	36–79	GBE + GT vs GT	20 mg/day	10 days	T,C
Tang 2021	30/30	61–87	GBE + GT vs GT	105 mg/day	10 days	T,C,A
Wang and Li 2008	36/36	35–51	GBE + GT vs GT	15 mL	14 days	T,C
Wang 2017	41/41	41–78	GBE + GT vs GT	70 mg/day	10 days	T,C
Wu and Run 2021	45/45	21–76	GBE + GT vs GT	25 mL	10 days	T,C,A
Xie 2018	60/60	70–88	GBE + GT vs GT	105 mg/day	10 days	T
Xin et al. 2020	85/85	18–70	GBE + GT vs GT	70 mg/day	14 days	T,C
Ye et al. 2018	69/69	24–68	GBE + GT vs GT	70 mg/day	10 days	T,C,A
Yu and Xu 2018	66/66	28–68	GBE + GT vs GT	6 ml	10 days	T,C,A
Zhang GJ et al. 2019	65/65	42–61	GBE + GT vs GT	105 mg/day	14 days	T,C,A
Zhang HJ et al. 2019	51/51	36–75	GBE + GT vs GT	87.5 mg/day	14 days	T,C,A
Zhang JY et al. 2019	40/39	41–72	GBE + GT vs GT	35 mg/day	10 days	T,A
Zhao et al. 2018	36/36	60–86	GBE + GT vs GT	70 mg/day	14 days	T,C

T: total effective rate; C: cure rate; A: adverse reaction; NT: not given; Con: control group; Exp: experimental group; GBE: *Ginkgo biloba* extract; GT: general treatments.

### Quality assessment

**Figure 2.**
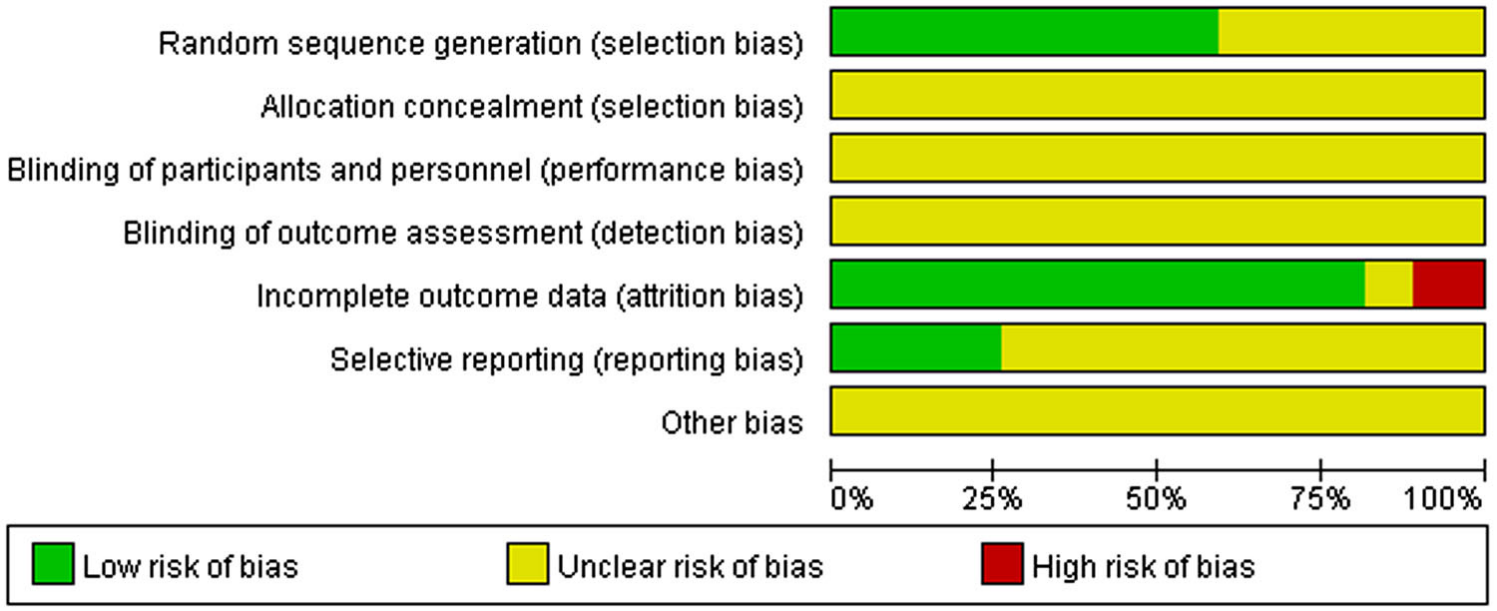
Risk of bias graph.

**Figure 3.**
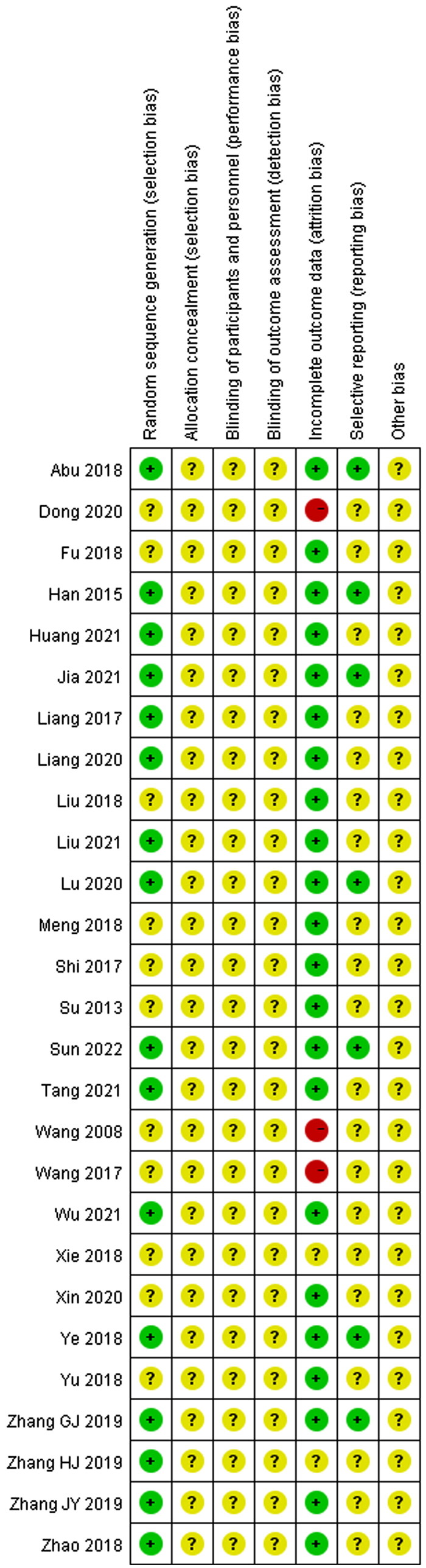
Risk of bias summary.

Among the 27 studies included, 16 studies (Han et al. 2015; Liang et al. 2017; Abu et al. 2018; Ye et al. 2018; Zhao et al. 2018; Zhang et al. 2019; 2019; 2019; Liang 2020; Lu et al. 2020; Huang and Luo 2021; Jia et al. 2021; Liu 2021; Tang 2021; Wu and Run 2021; Sun 2022) that describe details about the specific approaches of randomization were determined as low risk, while the remaining 11 studies (Wang and Li 2008; Su 2013; Shi 2017; Wang 2017; Fu 2018; Liu et al. 2018; Meng et al. 2018; Xie 2018; Yu and Xu 2018; Dong 2020; Xin et al. 2020) did not provide a clear randomization. None of these studies in this analysis provided a clear description of allocation concealment, performance bias, detection bias, and other biases. Moreover, 3 studies (Wang and Li 2008; Wang 2017; Dong 2020) were identified as high attrition risk due to the absence of outcome data, and no studies were considered as high reporting risk because the date was complete. The risk of bias assessment is shown in Figures 2 and 3.

### Outcome measures

#### Total effective rate

**Figure 4.**
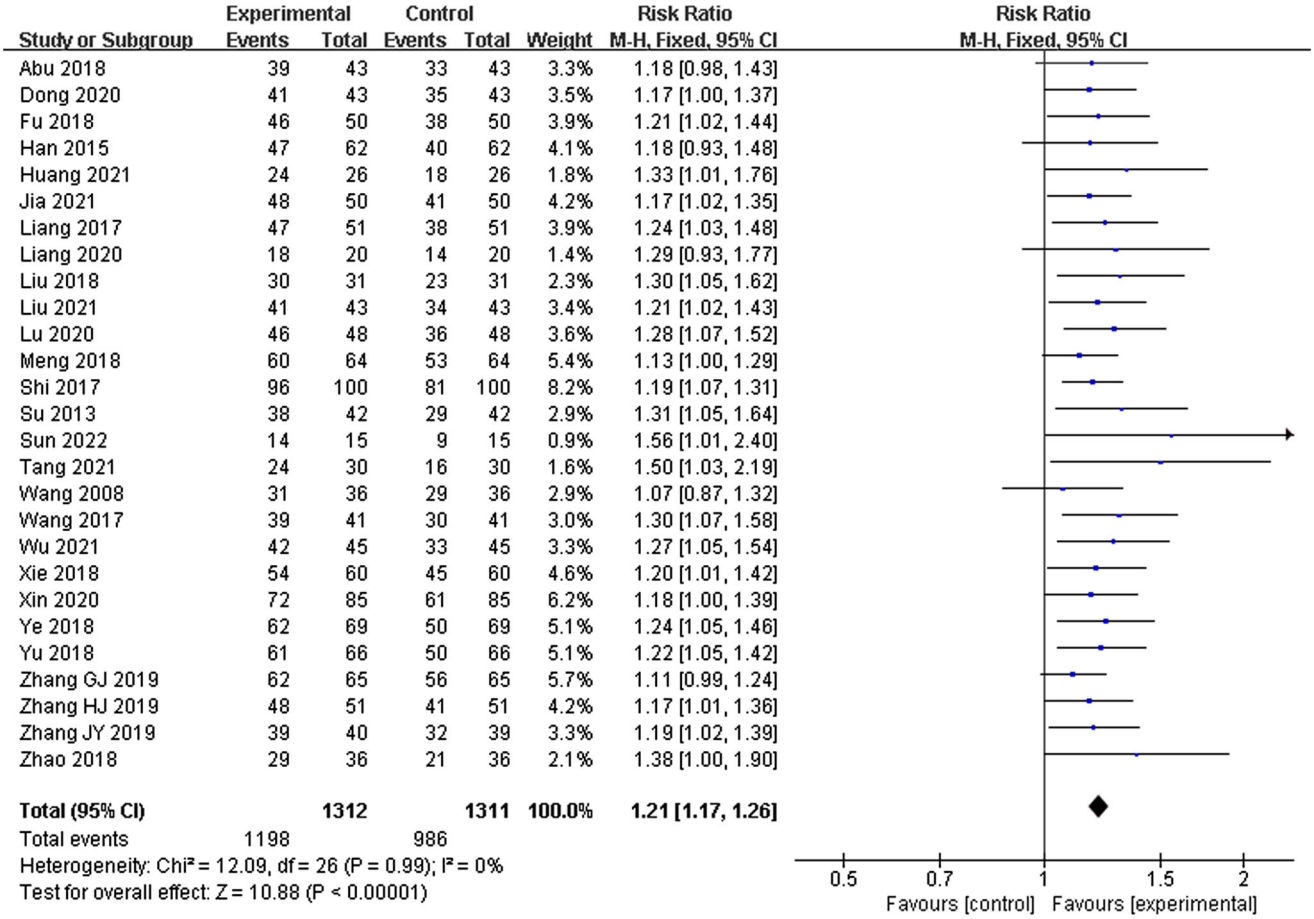
Total effective rate.

Twenty-seven studies were included in this section, with 1312 patients in the experimental group and 1311 patients in the control group. No statistical heterogeneity was shown (*p* = 0.99, *I*^2^ = 0%), and a fixed-effects model was used for meta-analysis. The results showed that there were significant differences between the experimental group and the control group. The patients in the experimental group showed a higher total effective rate than those in the control group (RR = 1.21, 95% CI: 1.17–1.26, *p* < 0.00001) (Figure 4).

#### Cure rate

**Figure 5.**
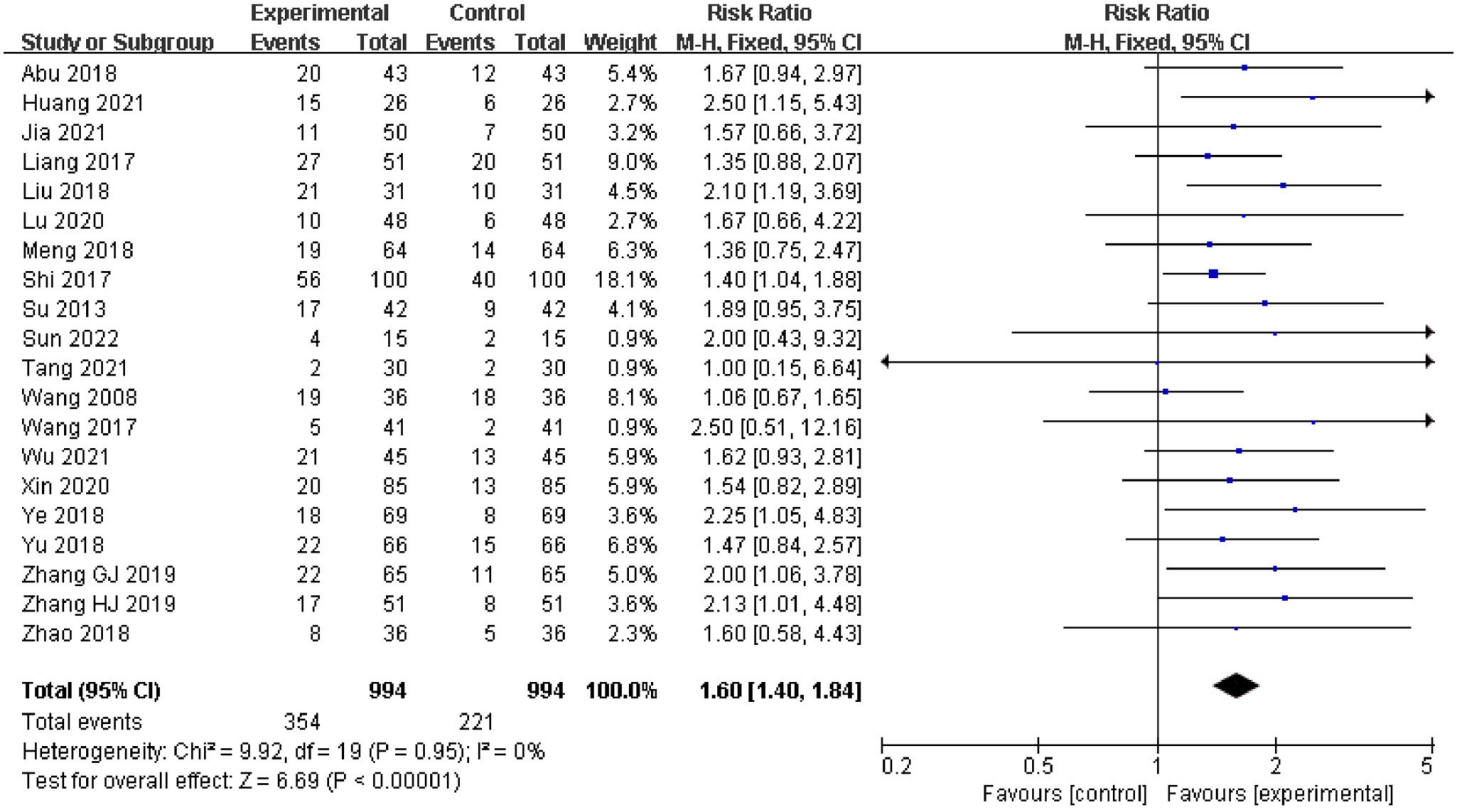
Cure rate.

Twenty studies (Wang and Li 2008; Su 2013; Liang et al. 2017; Shi 2017; Wang 2017; Abu et al. 2018; Liu et al. 2018; Meng et al. 2018; Ye et al. 2018; Yu and Xu 2018; Zhao et al. 2018; Zhang et al. 2019; Zhang et al. 2019; Lu et al. 2020; Xin et al. 2020; Huang and Luo 2021; Jia et al. 2021; Tang 2021; Wu and Run 2021; Sun 2022) were included, with 994 patients in the experimental group and 994 patients in the control group, while the remaining 7 studies were no cured data. A fixed-effect model was applied for meta-analysis as there was no statistical heterogeneity (*p* = 0.95, *I*^2^ = 0%). The results showed that there was a significant difference between the experimental group and the control group. The patients in the experimental group showed a better cure rate compared with the control group (RR = 1.60, 95% CI: 1.40–1.84, *p* < 0.00001) (Figure 5).

#### Pure tone hearing threshold

**Figure 6.**
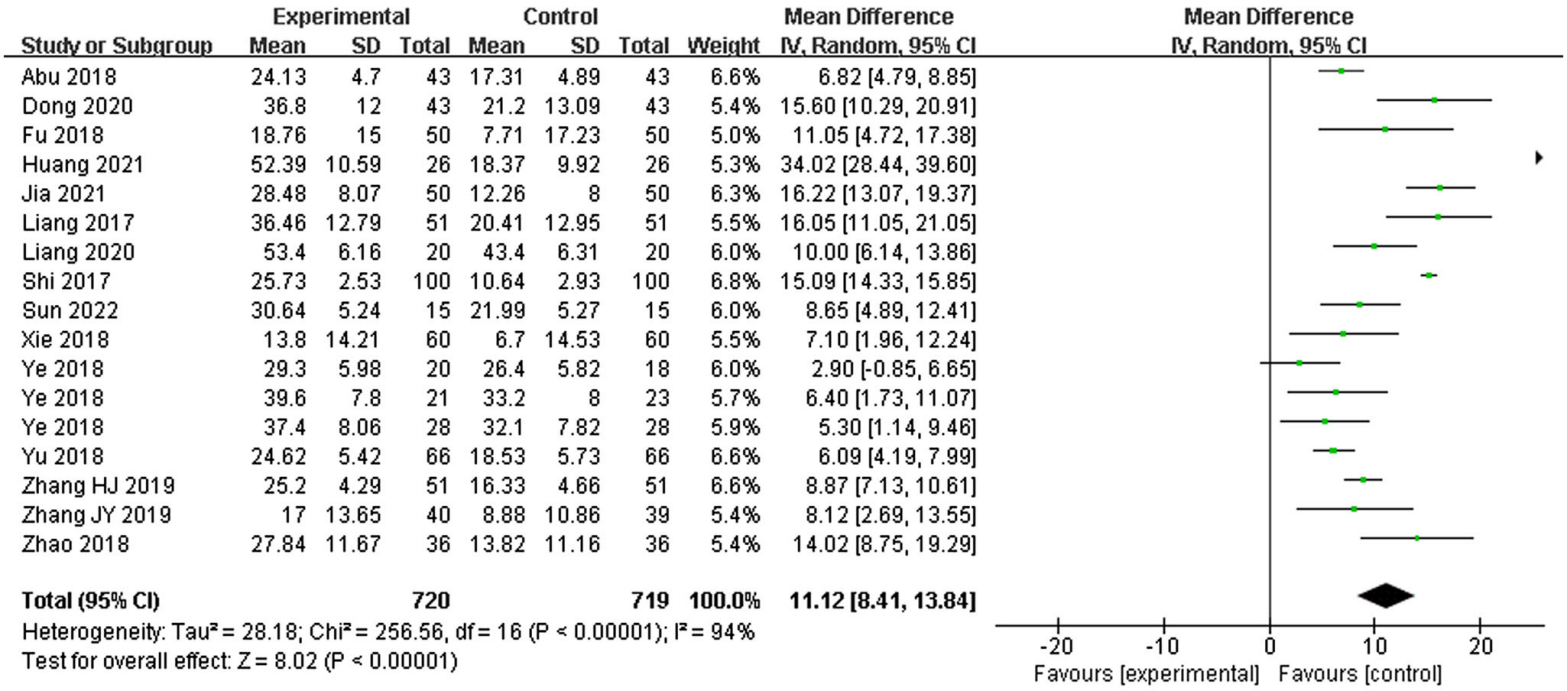
Pure tone hearing threshold.

Fifteen studies (Liang et al. 2017; Shi 2017; Abu et al. 2018; Fu 2018; Xie 2018; Ye et al. 2018; Yu and Xu 2018; Zhao et al. 2018; Zhang et al. 2019; 2019; Dong 2020; Liang 2020; Huang and Luo 2021; Jia et al. 2021; Sun 2022) were included, with 720 patients in the experimental group and 719 patients in the control group, while others were no pure tone hearing threshold data. A random-effects model was used to describe this indicator due to the high statistical heterogeneity between the two groups (*p* < 0.00001, *I*^2^ = 94%), according to the original data, pure tone hearing threshold improvement was calculated with standard deviation (SD). The experimental group could significantly improve the hearing results (*MD* = 11.12, 95% CI: 8.41–13.84, *p* < 0.00001) (Figure 6).

#### Hemorheology

Thirteen studies (Su 2013; Shi 2017; Abu et al. 2018; Meng et al. 2018; Ye et al. 2018; Zhao et al. 2018; Zhang et al. 2019; Dong 2020; Lu et al. 2020; Jia et al. 2021; Liu 2021; Tang 2021; Wu and Run 2021) were included, the remaining 14 articles were no information on hemorheology. Hemorheology assessments were conducted such as whole blood high shear viscosity, whole blood middle shear viscosity, whole blood low shear viscosity, plasma viscosity, whole blood viscosity, hematocrit (blood cells), fibrinogen and hematocrit (red blood cells) variations. Meta-analysis showed that GBE significantly improved hemorheology indexes in patients with SHL: whole blood high shear viscosity: *MD* = 1.46, 95% CI: 0.47–2.44, *p* = 0.004, whole blood medium shear viscosity: *MD* = 0.61, 95% CI: 0.36–0.6, *p* < 0.0001, whole blood low shear viscosity: *MD* = 2.54, 95% CI: 2.13–2.95, *p* < 0.0001, plasma viscosity: *MD* = 0.31, 95% CI: 0.27–0.36, *p* < 0.0001, whole blood viscosity: *MD* = 1.06, 95% CI: 0.8–1.31, *p* < 0.0001, hematocrit (blood cells): *MD* = 3.19, 95% CI: 2.15–4.24, *p* < 0.0001, fibrinogen: *MD* = 0.82, 95% CI: 0.47–1.16, *p* < 0.0001), while there was no significant difference as for the change of hematocrit (red blood cells) (*MD* = 4.15, 95% CI:-7.15–15.45, *p* = 0.47) (Table 2).

**Table 2.** Hemorheology index between experimental group and control group.

	No. of studies	Effects model	Heterogeneity		*MD*	95% CI	*p*
			*I*^2^ (%)	*p*			
Whole blood high shear viscosity	4	Random	97	*p* < 0.00001	1.46	(0.47, 2.44)	*p* < 0.00001
Whole blood medium shear viscosity	2	Fixed	0	*p* = 0.84	0.61	(0.36, 0.86)	*p* < 0.00001
Whole blood low shear viscosity	4	Fixed	0	*p* = 0.65	2.54	(2.13, 2.95)	*p* < 0.00001
Plasma viscosity	9	Fixed	40	*p* = 0.10	0.31	(0.27, 0.36)	*p* < 0.00001
Whole blood viscosity	4	Random	81	*p* = 0.001	1.06	(0.80, 1.31)	*p* < 0.00001
Hematocrit (blood cells)	4	Fixed	0	*p* = 0.65	3.19	(2.15, 4.24)	*p* < 0.00001
Hematocrit (red blood cells)	2	Random	97	*p* < 0.00001	4.15	(-7.15, 15.45)	*p* = 0.47
Fibrinogen	6	Random	91	*p* < 0.00001	0.82	(0.47, 1.16)	*p* < 0.00001

#### Adverse reactions

**Figure 7.**
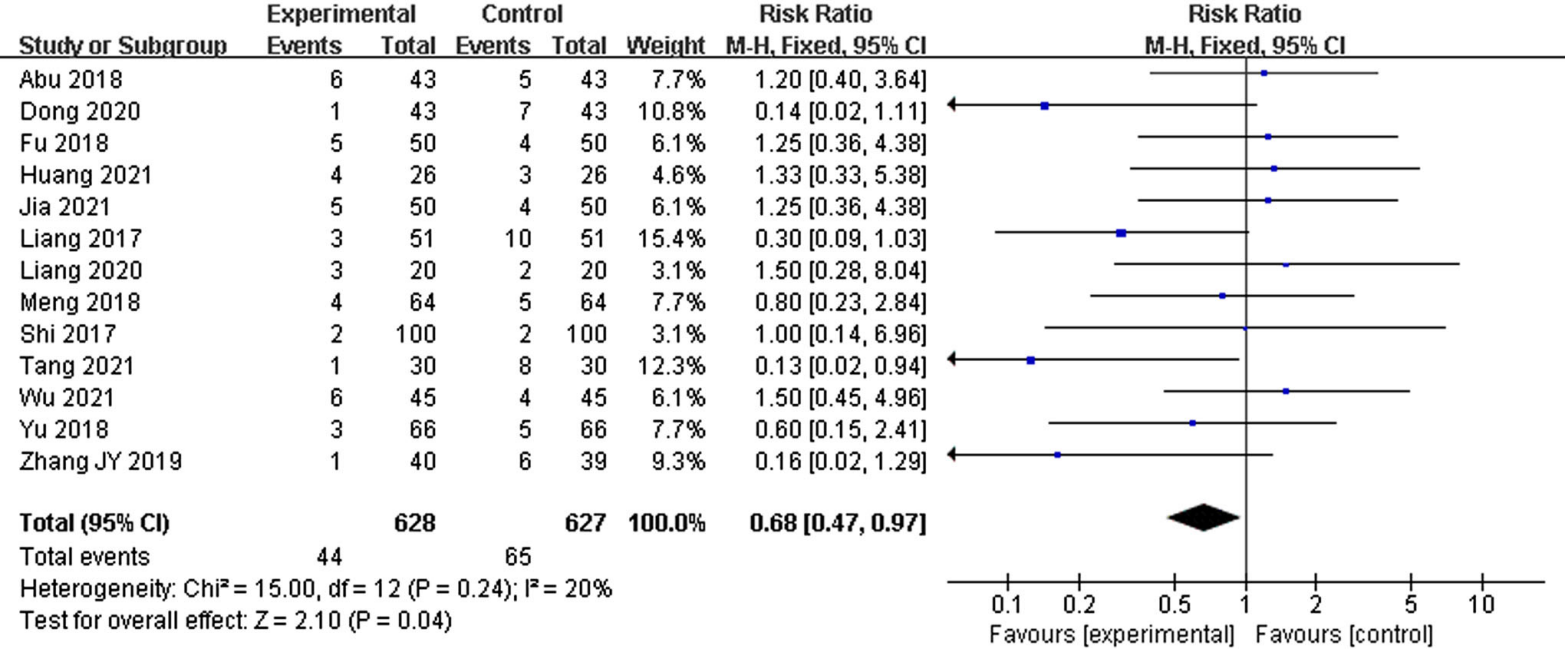
The number of adverse reactions.

Thirteen studies (Liang et al. 2017; Shi 2017; Abu et al. 2018; Fu 2018; Meng et al. 2018; Yu and Xu 2018; Zhang et al. 2019; Dong 2020; Liang 2020; Huang and Luo 2021; Jia et al. 2021; Tang 2021; Wu and Run 2021) reported ADR during the therapy, mainly manifested as nausea and vomiting, fever, digestive disorders, and sleep problems, but no serious adverse events were reported. Since the specific adverse reactions observed in each study were different and the number of the same adverse reactions was small, the assessments were not carried out in this analysis, however there was significant difference between two groups based on the number of adverse reactions (RR = 0.68, 95% CI: 0.47–0.97, *p* = 0.04) (Figure 7).

#### Subgroup analysis

Subgroup analysis was performed according to the route of administration, course of treatment, and GBE manufacturer. The information of the subgroups in this meta-analysis was listed as below: two subgroups of oral (Han et al. 2015; Liang et al. 2017; Shi 2017; Wang 2017; Abu et al. 2018; Meng et al. 2018; Xie 2018; Ye et al. 2018; Zhao et al. 2018; Zhang et al. 2019; 2019; 2019; Dong 2020; Lu et al. 2020; Xin et al. 2020; Huang and Luo 2021; Jia et al. 2021; Liu 2021; Tang 2021; Sun 2022) and injection (Wang and Li 2008; Su 2013; Fu 2018; Liu et al. 2018; Yu and Xu 2018; Liang 2020; Wu and Run 2021) according to the route of administration, two subgroups of 10 days (Han et al. 2015; Shi 2017; Wang 2017; Abu et al. 2018; Fu 2018; Liu et al. 2018; Meng et al. 2018; Xie 2018; Ye et al. 2018; Yu and Xu 2018; Zhang et al. 2019; Huang and Luo 2021; Jia et al. 2021; Tang 2021; Wu and Run 2021; Sun 2022) and 14 days (Wang and Li 2008; Liang et al. 2017; Zhao et al. 2018; Zhang GJ et al. 2019; Zhang HJ et al. 2019; Dong 2020; Liang 2020; Lu et al. 2020; Xin et al. 2020; Liu 2021) for the course of treatment, three subgroups for GBE manufacturers, Taiwan Jisheng Chemical Pharmaceutical Co., Ltd. in ten studies, YOUCARE Pharmaceutical Group Co., Ltd. in seven studies, while no specific information of GBE was described in seven studies. Since there was no heterogeneity among the subgroups, a fixed effect model (*p* > 0.1, *I*^2^ = 0%) was applied. The results showed that GBE can improve the total effective rate of the treatment on SHL with different dosages, courses of treatment and manufacturers compared with the control group (RR = 1.21, 95% CI: 1.17–1.26, *p* < 0.00001; RR = 1.23, 95% CI: 1.14–1.33, *p* < 0.00001; RR = 1.22, 95% CI: 1.17–1.28, *p* < 0.00001; RR = 1.20, 95% CI: 1.14–1.27, *p* < 0.00001; RR = 1.20, 95% CI: 1.13–1.26, *p* < 0.00001; RR = 1.27, 95% CI: 1.17–1.37, *p* < 0.0000; RR = 1.19, 95% CI: 1.12–1.27, *p* < 0.00001) (Table 3).

**Table 3.** The results of subgroup analysis.

	No. of studies	Effects model	Heterogeneity		*MD*	95% CI	*p*
			*I*^2^ (%)	*p*			
Route S. (Total)	27	Fixed	0	*p* = 0.99	1.21	(1.17, 1.26)	*p* < 0.00001
Oral	20	Fixed	0	*p* = 0.97	1.21	(1.16, 1.26)	*p* < 0.00001
Injection	7	Fixed	0	*p* = 0.86	1.23	(1.14, 1.33)	*p* < 0.00001
Course S. (Total)	26	Fixed	0	*p* = 0.99	1.21	(1.17, 1.25)	*p* < 0.00001
10d	16	Fixed	0	*p* = 0.98	1.22	(1.17, 1.28)	*p* < 0.00001
14d	10	Fixed	0	*p* = 0.83	1.19	(1.13, 1.26)	*p* < 0.00001
GBE manufacturer S. (Total)	24	Fixed	0	*p* = 0.98	1.21	(1.17, 1.26)	*p* < 0.00001
TJCP	10	Fixed	0	*p* = 0.99	1.20	(1.13, 1.26)	*p* < 0.00001
YCPG	7	Fixed	0	*p* = 0.84	1.27	(1.17, 1.37)	*p* < 0.00001
NSIG	7	Fixed	0	*p* = 0.54	1.19	(1.17, 1.26)	*p* < 0.00001

Route S.: route subgroup; Course S.: course subgroup; GBE manufacturer S.: GBE manufacturer subgroup; TJCP: Taiwan Jisheng Chemical Pharmaceutical Co., Ltd. YCPG: YOUCARE Pharmaceutical Group Co., Ltd. NSIG: No specific information of GBE

### Publication bias

**Figure 8.**
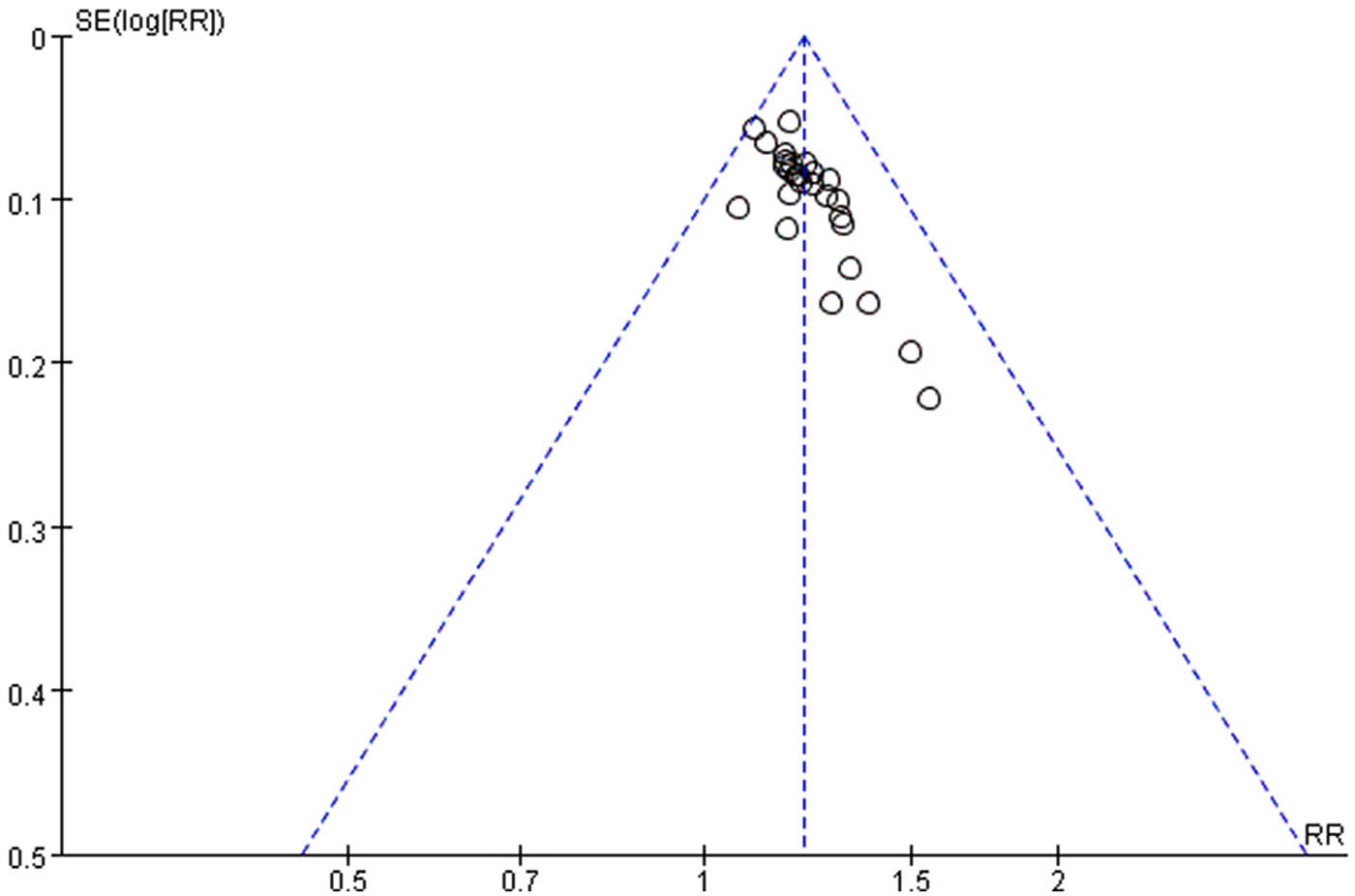
Funnel plot of total effective rate.

**Figure 9.**
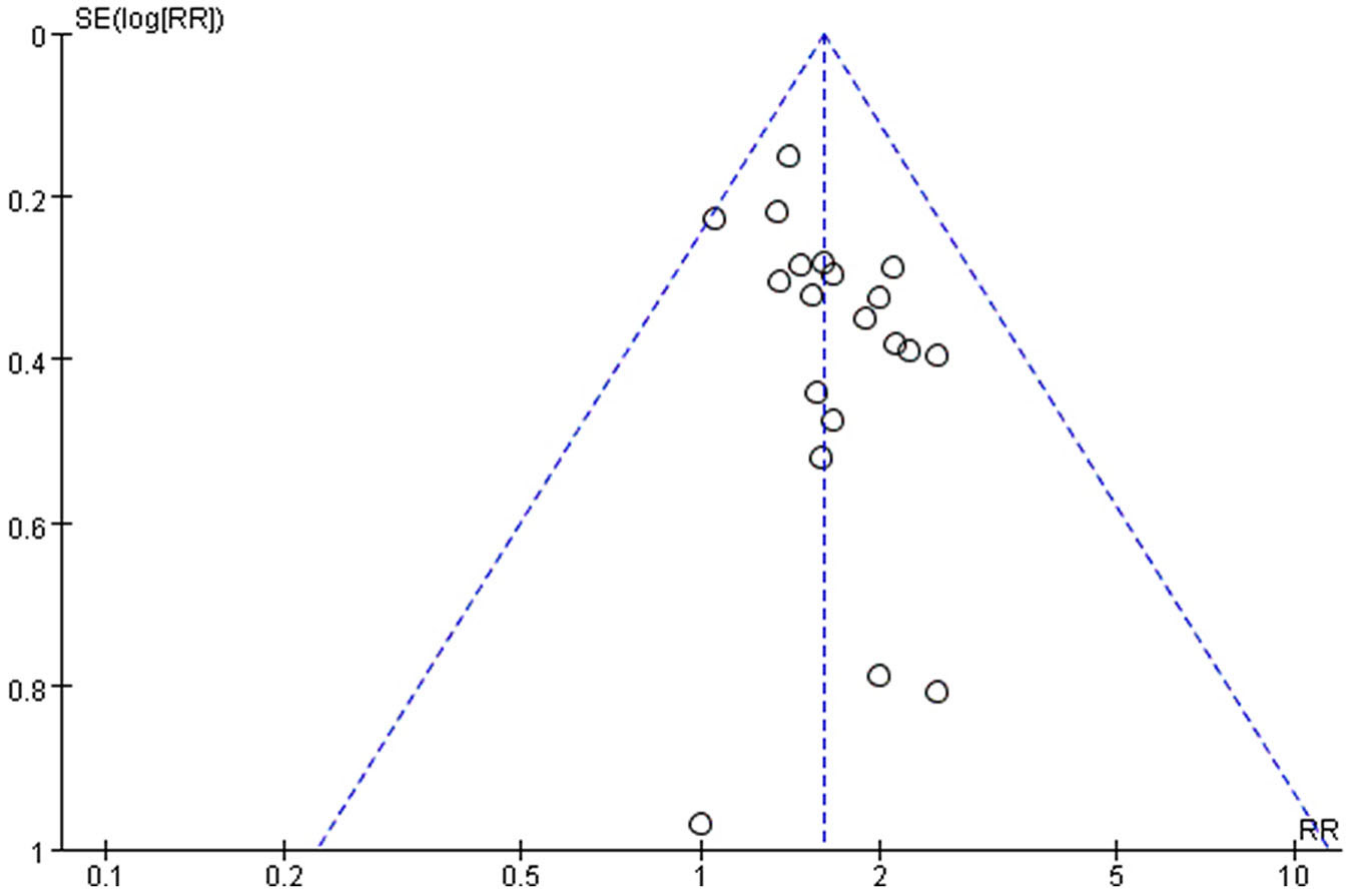
Funnel plot of cure rate.

**Figure 10.**
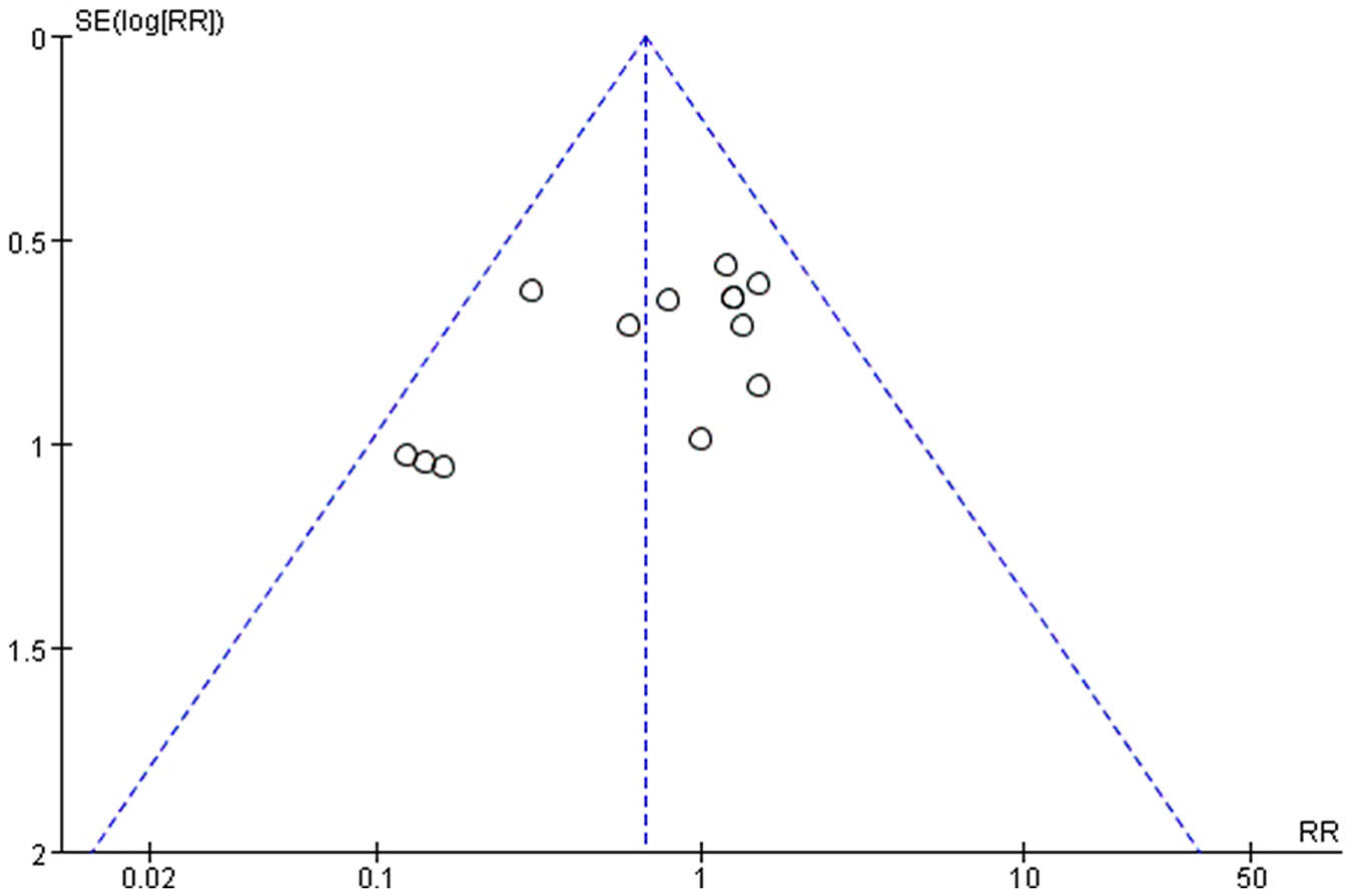
Funnel plot of adverse reactions.

In order to evaluate the publication bias that might be produced in this meta-analysis, funnel plots and Egger’s test were examined. The results showed that the funnel plots of the total effective rate, cure rate and adverse reactions were asymmetrically distributed (Figures 8–10), suggesting that the publication bias may exist, and Egger’s test (*p* < 0.0001) also indicated the possible existence of publication bias.

## Discussion

SHL is sensorineural hearing loss with the characteristics of sudden loss or even disappearance. But the pathogenesis of SHL has not been clear until now. It may cause permanent deafness without timely treatment, which leads to great distress or inconvenience to patients. It is reported that inner ear microcirculation disturbance, insufficient blood supply, viral infection, immune imbalance, and rupture of the membrane labyrinth may be correlated with the pathology of SHL (He 2018; Liu and Zhang 2018; Compagnone et al. 2022; Khasanov et al. 2022). Excessive social pressure and negative life events are important factors inducing sudden deafness, so psychological factors are also one of the potential factors of sudden deafness. In addition, ischemia, hypoxia and microcirculatory disorders in inner ear can also lead to vasospasm, thrombosis, increased blood viscosity, slow hemodynamic changes, increased oxygen free radicals or decreased free radical scavenging enzyme activity, leading to cochlear receptor ischemia, spiral nerve degeneration, and ultimately hearing impairment. Many viruses, such as measles virus, herpes simplex virus and cytomegalovirus, can incubate in the nervous system. The herpes simplex virus can infect the auditory pathway and nerve, especially through blood circulation into the inner ear, inducing cochlear microcirculation disorders and sudden deafness (Kuhn et al. 2011; Chen et al. 2019).

GBE is a traditional Chinese medicine preparation extracted from *Ginkgo biloba* leaves. The main active ingredients contain ginkgolides and flavonoid glycosides, which can inhibit the formation of ear thrombus, scavenge oxygen free radicals, improve vascular microcirculation, and reduce blood viscosity (Wang and Yang 2001; Wang and Li 2009; Zhao 2010; Barth et al. 2021). It can effectively promote inner ear microcirculation, help patients recover from hearing dysfunction, accelerate the relief of tinnitus and vertigo, which exerts significant pharmacological activity on SHL. Recent studies have shown that ginkgolides contained in ginkgo leaf preparations are antagonists of platelet activating factor (PAF) (Smith et al. 1996), which can prevent platelet aggregation and thrombosis induced by PFA, reduce blood viscosity and improve blood rheology. GBE exerts significant antioxidant activity (Singh et al. 2019; Bohlken et al. 2022), which can improve the activity of superoxide dismutase (Barth et al. 2021), accelerate the scavenging of oxygen free radicals caused by ischemia, and protect cell tissues. GBE also has vasodilator effect, it can stimulate the production of endothelium-derived relaxing factor and prostacyclin, promote the relaxation of vascular smooth muscle, which could maintain good arteriovenous tension, and increase the blood flow in and around the injury spot (Tian et al. 2017). GBE dilates the auricular arterioles, increases the blood flow of the inner ear, enhances the compensatory function of the inner ear, improves tissue metabolism, alleviates labyrinthine artery edema, and accelerates the disappearance of clinical symptoms (Spiegel et al. 2018).

A large number of randomized controlled trials from China had been reviewed, while the sample size of single trial was small. Moreover, the evidence for the clinical application was weak. Therefore, this meta-analysis was conducted to systematically evaluate the total effective rate and cure rate of GBE on SHL with randomized controlled trials to provide a scientific basis for clinical practice. 2685 cases were analyzed in this study and the results showed that GBE was statistically significant (*p* < 0.01) in the total effective rate (RR = 1.22, 95% CI: 1.18–1.26, *p* < 0.00001), cure rate (RR = 1.60, 95% CI: 1.40–1.84, *p* < 0.00001), pure tone hearing threshold improvement (*MD* = 11.12, 95% CI: 8.41–13.84, *p* < 0.00001), hemorheological indicators (whole blood high shear viscosity: *MD* = 1.46, 95% CI: 0.47–2.44, *p* = 0.004, whole blood medium shear viscosity: *MD* = 0.61, 95% CI: 0.36–0.6, *p* < 0.0001, whole blood low shear viscosity: *MD* = 2.54, 95% CI: 2.13–2.95, *p* < 0.0001, plasma viscosity: *MD* = 0.31, 95% CI: 0.27–0.36, *p* < 0.0001, whole blood viscosity: *MD* = 1.06, 95% CI: 0.8–1.31, *p* < 0.0001, hematocrit (blood cells): *MD* = 3.19, 95% CI: 2.15–4.24, *p* < 0.0001, fibrinogen: *MD* = 0.82, 95% CI: 0.47–1.16, *p* < 0.0001), and lower incidence rate of adverse reactions (RR = 0.68, 95% CI: 0.47–0.97, *p* = 0.04). In addition, according to subgroup analysis, GBE can be administered orally or by injection, although the metabolisms are different, SHL can be significantly improved regardless of the administration route (oral: RR = 1.21, 95% CI: 1.17–1.26, *p* < 0.00001; Injection: RR = 1.23, 95% CI: 1.14–1.33, *p* < 0.00001).

Our analysis showed that GBE can improve the total effective rate and cure rate, it was also reported that GBE had been applied to SHL and exerted a strong effect (Burschka et al. 2001; Si, Yu, et al. 2022), with pure tone hearing threshold improved significantly, it was consistent with our results. In the early studies (Spiegel et al. 2018; Radunz et al. 2020; Barth et al. 2021), GBE had an obvious effect in relieving tinnitus and improving cochlear blood flow, while our meta-analysis showed that GBE can improve pure tone hearing threshold and hemorheology, our results are in line with the former studies. Therefore, treatment of SHL with GBE adjuvant can be practicable because of its efficacy and safety.

However, this study has some limitations. First, some literature does not report specific randomized control trials, which may cause random sequence generation bias, the details were insufficient, such as randomization methods, allocation concealment, performance bias, attrition bias, reporting bias, some literature had high risk factors in quality evaluation, so it has a certain impact on the strength of the evidence. Second, the literature included in this study is restricted to the region of China, which may cause ethnic and regional bias. Finally, no long-term prognosis (such as overall survival, progression-free survival and recurrence rate) was reported in the literature, making it difficult to analyze the overall safety and efficacy. Therefore, large-scale, multi-center, randomized, double-blind and other high-quality clinical randomized trials are in demand in the future (Moher et al. 2009), and combining the characteristics of GBE in terms of dosage, duration of treatment, and incidence rate of adverse reactions, and comprehensively consider long-term treatment effects and drug safety and effectiveness, so as to provide strong evidence for further clinical practice.

## Conclusion

The results of our meta-analysis showed that adjuvant therapy with GBE may be better than no GBE, the total effective rate and cure rate are significantly improved, the pure tone hearing threshold and hemorheology indexes are significantly improved with treatment, and the rate of adverse reactions is reduced. Based on the results of our analysis and the theoretical basis of SHL, GBE may be a perfect complementary and alternative therapy strategy. However, the low quality of some articles resulted in the potential risk of bias, which affected the reliability of this study to some extent. Therefore, the long-term efficacy and safety of GBE on SHL still need to be verified by large multicenter and carefully designed rigorous RCTS to provide reliable evidence for the efficacy of GBE as an adjunct on SHL.
